# Plasma proteomics shows an elevation of the anti-inflammatory protein APOA-IV in chronic equine laminitis

**DOI:** 10.1186/1746-6148-8-179

**Published:** 2012-09-27

**Authors:** Samantha M Steelman, Bhanu P Chowdhary

**Affiliations:** 1 Veterinary Integrative Biosciences, College of Veterinary Medicine, Texas A&M University, College Station, TX, 77845-4458, USA

## Abstract

**Background:**

Equine laminitis is a devastating disease that causes severe pain in afflicted horses and places a major economic burden on the horse industry. In acute laminitis, the disintegration of the dermal-epidermal junction can cause the third phalanx to detach from the hoof wall, leaving the horse unable to bear weight on the affected limbs. Horses that survive the acute phase transition into a chronic form of laminitis, which is often termed “founder”. Some evidence suggests that chronic laminar inflammation might be associated with alterations in the endocrine and immune systems. We investigated this broad hypothesis by using DIGE to assess global differences in the plasma proteome between horses with chronic laminitis and controls.

**Results:**

We identified 16 differentially expressed proteins; the majority of these were involved in the interrelated coagulation, clotting, and kininogen cascades. Clinical testing of functional coagulation parameters in foundered horses revealed a slight delay in prothrombin (PT) clotting time, although most other indices were within normal ranges. Upregulation of the intestinal apolipoprotein APOA-IV in horses with chronic laminitis was confirmed by western blot.

**Conclusions:**

Our results support the hypothesis that localized laminar inflammation may be linked to systemic alterations in immune regulation, particularly in the gastrointestinal system. Gastrointestinal inflammation has been implicated in the development of acute laminitis but has not previously been associated with chronic laminitis.

## Background

Laminitis is a painful and debilitating disease of the equine foot. The specific molecular factors initiating laminitis are unknown, but it is often associated with insulin resistance and obesity and can be precipitated by diseases such as colic and diarrhea [1,2]. The acute phase of the disease is characterized by the onset of severe lameness and disintegration of the laminae, which connect the hoof wall to the underlying dermis and third phalanx [3]. In many cases, the pathology progresses until the weight of the horse causes dorsopalmar rotation of the third phalanx, detaching it from the hoof wall. This condition is usually not reversible and euthanasia is often the only humane option. In many instances, horses that survive an episode of acute laminitis are crippled for life. This stage of the disease is termed chronic laminitis or “founder”. Acute laminitis transitions to the chronic form of the disease in an estimated 75% of cases [4], leaving the majority of afflicted horses permanently lame.

Despite intense efforts to understand the root cause of acute laminitis, much less attention has been focused on the pathophysiology of chronic laminitis. A handful of studies have investigated changes in laminar morphology, metabolism, and gene expression in foundered horses [5-8], although several recent studies have focused on the efficacy of different management strategies to ameliorate pain and improve quality of life [9,10]. Little is known, therefore, about how chronic foot pain and inflammation affect the horse on a global level. A recent study quantified behavioral changes indicative of pain in horses with chronic laminitis; these changes were associated with alterations in morphology and gene expression of the lateral digital nerve and dorsal root ganglia that are consistent with neuropathic pain, suggesting that some of the pain associated with chronic laminitis results from peripheral nerve damage outside of the foot [11]. In addition, Wagner et al. found that horses with chronic laminitis show excessive dermal inflammatory responses to a panel of allergens [12], suggesting an inappropriate activation of the systemic immune response. To our knowledge, however, these studies are the only published reports describing basic scientific investigations of chronic laminitis.

The dearth of information regarding how chronic laminar inflammation affects the horse as a whole is a major obstacle to understanding why certain horses experience recurrent bouts of laminitis and how these bouts can be prevented. As a first step towards characterizing global alterations in the physiology of horses with chronic laminitis, we used DIGE followed by LC-MS/MS to detect differentially expressed plasma proteins in foundered horses. Plasma was chosen as the sample matrix because the blood is a primary means of transport for numerous effector hormones and cytokines of the immune and endocrine systems, which have been suggested to be altered in chronic laminitis [12,13].

## Results

### Population characteristics

The control (CON) and laminitis (LMN) groups were balanced for age and gender. None of the horses in the CON group exhibited signs of lameness at the time of sample collection. Three of the four horses in the LMN group were persistently lame; the fourth experienced intermittent lameness but was sound on the day of sample collection. Two of the permanently lame horses were being treated with a low level of phenylbutazone (1 g per day), a non-steroidal anti-inflammatory drug commonly used in veterinary practice. Horses in both groups were in good body condition and did not exhibit any evidence of injury or illness other than laminitis. White blood cell counts obtained on the same day that plasma was collected for DIGE were within normal parameters and did not differ between groups (P = 0.46).

### Detection of differentially expressed spots

**Figure 1:**
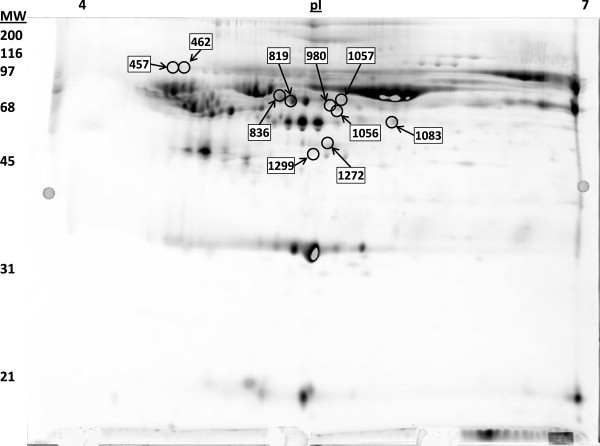
** Image of the master DIGE gel stained with Deep Purple showing equine plasma depleted of albumin and IgG.** The location of each of the ten spots chosen for identification with LC-MS/MS is labelled with the corresponding spot number.

In order to view global changes in the plasma of horses with chronic laminitis, we performed DIGE on plasma depleted of albumin and IgG. The Decyder software detected ~2500 individual spots, 42 of which were differentially expressed in the LMN group as compared to controls (P < 0.05). Manual inspection of the spot histograms in the DIA module revealed that 32 of the 42 spots were most likely artefacts based on the size and shape of the 3D intensity profile. Ten spots (maximum slope < 2.5, area > 300 pixels, and maximum volume > 100,000 pixels) were chosen for identification using LC-MS/MS (Figure 1 and Table 1). Table 1 describes the relative fold change in intensity and associated P value for each of the 10 spots. We found no evidence to suggest that the horse in the LMN group that was not lame at the time of sampling differed from the other horses in the group or was a possible outlier. In addition, no differences were noted between the two horses treated with phenylbutazone and those that were unmedicated.

**Table 1:** Fold changes and P values of spots selected for protein identification

**Spot #**	**Fold Change**	**P value**
457	−1.26	0.027
462	−1.2	0.005
819	−1.3	0.031
836	−1.75	0.043
980	1.49	0.036
1056	−1.37	0.028
1057	−1.34	0.021
1083	−1.67	0.036
1272	−2.11	0.002
1299	2.26	0.006

### Protein identification

Twenty one proteins were identified from the LC-MS/MS data generated from the 10 digested spots (Table 2). Human keratin and trypsin derivatives were assumed to be contaminants (trypsin was used as a digestion enzyme prior to LC-MS/MS) and not included in the final list of differentially expressed proteins. Likewise, although the majority of plasma albumin was depleted during sample preparation, it was identified in one of the differentially expressed spots but was not included in Table 2. Proteins identified from spots upregulated in LMN horses included angiotensinogen, alpha-2-macroglobulin, and apolipoprotein A-IV; these are presented at the top of Table 2. Antithrombin and complement factor C3 precursor were identified in both up- and down-regulated spots. The remainder of the differentially expressed proteins were from spots downregulated in the LMN group.

**Table 2:** Names and NCBI accession numbers of proteins identified in differentially expressed spots

**DIGE Spot #**	**Protein name**	**Accession number (NCBI)**	**MW (kDa)**	**Sequence coverage (%)**	**No. peptides**
***Upregulated***					
**980**	PREDICTED: similar to angiotensinogen [Equus caballus]	gi|194206059	70	3.12%	2
**980**	PREDICTED: alpha-2-macroglobulin isoform 3 [Macaca mulatta]	gi|109095556	161	1.31%	2
**980**	PREDICTED: similar to antithrombin protein [Equus caballus]	gi|149708147	52	5.40%	3
**1299**	PREDICTED: apolipoprotein A-IV [Equus caballus]	gi|149716543	43	6.82%	3
**1299**	PREDICTED: similar to Complement C3 precursor [Equus caballus]	gi|194212541	186	2.05%	4
***Dowregulated***					
**457**	PREDICTED: similar to alpha-1-antitrypsin; serine protease inhibitor [Equus caballus]	gi|194225326	47	13.30%	5
**457**	PREDICTED: similar to Alpha-2-HS-glycoprotein precursor (Fetuin-A) [Equus caballus]	gi|194222675	39	10.20%	3
**457**	alpha-1-antitrypsin [Equus caballus]	gi|197631767	47	11.90%	6
**462**	PREDICTED: similar to Plasma protease C1 inhibitor precursor (C1 Inh) (C1-inhibiting factor) [Equus caballus]	gi|149758084	53	9.24%	5
**819**	PREDICTED: similar to Vitronectin precursor (S-protein) (V75) [Equus caballus]	gi|194217342	55	3.51%	2
**819**	PREDICTED: similar to Plasma protease C1 inhibitor precursor (C1 Inh) (C1-inhibiting factor) [Equus caballus]	gi|149758084	53	5.88%	3
**819**	PREDICTED: kininogen 1 [Equus caballus]	gi|149731462	58	5.83%	3
**836**	PREDICTED: similar to Fibrinogen gamma chain [Equus caballus]	gi|194208381	52	4.20%	2
**836**	immunoglobulin alpha constant heavy chain [Equus caballus]	gi|32331167	37	5.83%	2
**836**	PREDICTED: similar to antithrombin protein [Equus caballus]	gi|149708147	52	19.40%	8
**1056**	Immunoglobulin mu heavy chain constant chain secreted form	gi|51831151	50	4.43%	2
**1056**	PREDICTED: similar to Fetuin-B precursor (Gugu) [Equus caballus]	gi|149731458	42	7.59%	3
**1056**	PREDICTED: similar to Fibrinogen gamma chain [Equus caballus]	gi|194208381	52	11.70%	7
**1057**	PREDICTED: similar to Fetuin-B precursor (Gugu) [Equus caballus]	gi|149731458	42	8.12%	3
**1057**	PREDICTED: similar to Fibrinogen gamma chain [Equus caballus]	gi|194208381	52	11.30%	5
**1083**	PREDICTED: similar to Alpha-2-antiplasmin precursor (Alpha-2-plasmin inhibitor) [Equus caballus]	gi|149724158	55	8.96%	4
**1083**	PREDICTED: similar to Fibrinogen gamma chain [Equus caballus]	gi|194208381	52	7.08%	3
**1083**	PREDICTED: similar to Fetuin-B precursor (Gugu) [Equus caballus]	gi|149731458	42	17.00%	6
**1272**	PREDICTED: similar to coagulation factor X protein [Equus caballus]	gi|194222063	54	5.77%	3
**1272**	PREDICTED: similar to Complement C3 precursor [Equus caballus]	gi|194212541	186	1.99%	3

Predicted molecular weight of each protein, the number of peptides identified from each protein, and the percentage of the total sequence covered by the identified peptides are also shown.

### Annotation and ontology

The horse genome, though sequenced, is only partially annotated. Therefore, the human orthologs were used to identify gene function and overrepresented gene ontologies among differentially expressed proteins (Table 3). Five of the proteins identified by MS were found in multiple spots and one protein showed differential expression of two of its subunits (fibrinogen beta and gamma chains). Therefore, of the 21 proteins, only 16 were unique. All 16 proteins belonged to the extracellular compartment or were secretory molecules stored within platelet alpha granules. Molecular functions of the proteins were predominated by serine endopeptidase inhibitory activity. Overrepresented biological process ontologies included response to wounding, defense response, coagulation, and inflammatory response. Eleven of the 16 proteins were identified as being involved in the inter-related coagulation, kininogen, and complement cascades. The remaining proteins included alpha-2-HS glycoprotein (fetuin A) and fetuin B, both molecular chaperones and participants in the acute phase response [14,15], the immunoglobulin heavy chains alpha and mu (IgA and IgM), and apolipoprotein A-IV (APO AIV), which is an anti-inflammatory molecule [16]. 

**Table 3:** Gene ontologies of the human orthologs of differentially expressed equine plasma proteins

**Spot**	**Gene Name**	**Expression**	**Molecular Function**	**KEGG Pathway**
**980**	alpha-2-macroglobulin	Up-regulated	Serine-type endopeptidase inhibitor activity, growth factor binding, cytokine binding, chemokine binding	Complement and coagulation cascades
**980**	angiotensinogen	Up-regulated	Serine-type endopeptidase inhibitor activity, growth factor activity, acetyltransferase activator activity, angiotensin receptor binding	Renin-angiotensin system
**1299**	apolipoprotein A-IV	Up-regulated	Phospholipid binding, antioxidant activity, cholesterol transporter activity	
**1272**	coagulation factor X	Down-regulated	Serine-type endopeptidase activity	Complement and coagulation cascades
**836**	fibrinogen beta chain	Down-regulated	Protein binding, bridging	Complement and coagulation cascades
**1056, 1057, 1083**	fibrinogen gamma chain	Down-regulated	Protein binding, bridging	Complement and coagulation cascades
**836**	IgM	Down-regulated	Antigen binding	
**1056**	IgA	Down-regulated	Antigen binding	
**819**	kininogen 1	Down-regulated	Pattern binding, cysteine-type endopeptidase inhibitor activity, heparin binding	Complement and coagulation cascades
**819**	protein S, alpha (vitronectin)	Down-regulated	Endopeptidase inhibitor activity, calcium ion binding	Complement and coagulation cascades
**457**	alpha-1 antitrypsin	Down-regulated	Serine-type endopeptidase inhibitor activity	Complement and coagulation cascades
**836, 980**	antithrombin	Down-regulated (836), Up-regulated (980)	Pattern binding, serine-type endopeptidase inhibitor activity, heparin binding	Complement and coagulation cascades
**1083**	alpha-2 antiplasmin	Down-regulated	Serine-type endopeptidase inhibitor activity	Complement and coagulation cascades
**462, 819**	C1 inhibitor	Down-regulated	Complement binding, serine-type endopeptidase inhibitor activity	Complement and coagulation cascades
**457**	alpha-2-HS-glycoprotein	Down-regulated	Cysteine-type endopeptidase inhibitor activity, protein tyrosine kinase inhibitor activity	
**1056, 1057, 1083**	fetuin B	Down-regulated	Cysteine-type endopeptidase inhibitor activity	
**1272, 1299**	similar to Complement C3 precursor	Down-regulated (1272), Up-regulated (1299)	Endopeptidase inhibitor activity	Complement and coagulation cascades

### Immunoglobulin concentrations

The differences in IgA and IgM concentrations suggested by DIGE were not confirmed by ELISA. IgA concentrations were similar between CON and LMN groups (266 ± 44 mg/dL LMN vs. 254 ± 44 mg/dL CON, P = 0.45), as were IgM concentrations (107 ± 11 mg/dL LMN vs. 115 ± 7 mg/ml CON, P = 0.27).

### Coagulation panel

Because the majority of differentially expressed proteins were involved in the coagulation cascade, we assessed the physiological relevance of our DIGE results by testing coagulation parameters in seven LMN horses: another three horses with chronic laminitis were used in addition to the four horses used for the DIGE experiment. Five of the seven horses, including three of the four used for the DIGE study, had prolonged prothrombin times (PT, Table 4), indicating that the LMN group was statistically different from the reference population (P < 0.01). It should be noted that none of the animals in the LMN group had a history of coagulopathy. Fibrinogen concentration derived from prothrombin time was elevated in six of seven animals, including all four horses used for the DIGE experiment, although this finding was not corroborated by the more accurate Clauss method of fibrinogen quantification. Fibrin D-dimer was elevated in only one animal.

**Table 4:** Coagulation indices in horses with chronic laminitis

**Horse ID**	**PT**	**PTT**	**AT**	**Fib PT**	**Fib Clauss**	**D dimer**
L1	10.8	44.2	258	538*	232	618*
L2	11.3*	48.9	222	580*	209	211
L3	11.5*	46.3	216	543*	199	212
L4	11.0	34.0*	238	378	168	261
L5	11.6*	49.6	228	452*	195	497
L7	11.5*	45.3	206	783*	311*	238
L8	11.3*	42.9	226	396*	184	413

Normal clinical reference ranges are as follows: PT: prothrombin time, 9.7 – 11.2 s. PTT: partial thromboplastin time, 39.3 – 56.1 sec. AT: antithrombin activity, >130 % NHP. Fib PT: fibrinogen (PT method), 207–386 mg/dL. Fib Clauss: fibrinogen (Clauss method), 96–271 mg/dL. D dimer: D dimer of fibrin, 111 – 612 ng/ml. Asterisks indicate deviation from normal reference range.

### APOA-IV concentration

**Figure 2:**
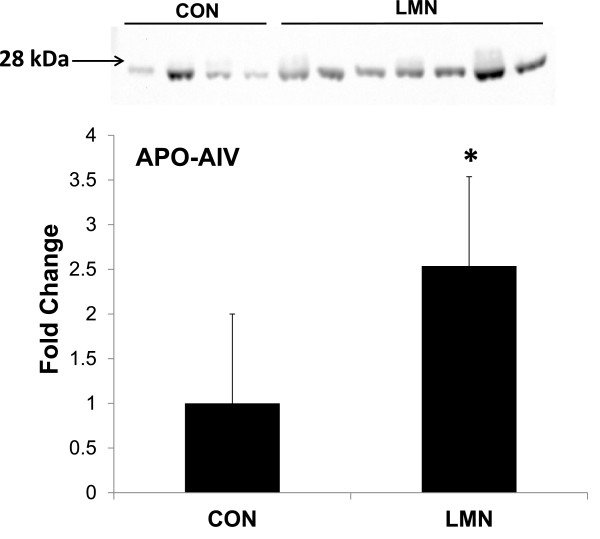
Western blot and densitometry quantification of bands shows a significant (P < 0.05) elevation of apolipoprotein A-IV in horses with chronic laminitis (LMN) as compared to normal controls (CON).

Immunoblotting for equine APOA-IV using an anti-human antibody was successful, with a single band detected at ~30 kDa. No other bands were detected on the blot [17]. Densitometry showed a significant (P < 0.05) elevation of APOA-IV in horses with chronic laminitis (Figure 2). 

## Discussion

The data presented herein describe, for the first time, plasma proteome changes in chronic equine laminitis. Our results suggest that a number of proteins involved in immune regulation are differentially expressed in horses with chronic equine laminitis. In particular, the anti-inflammatory protein APOA-IV was elevated approximately two-fold in foundered horses. Although no major deficits in the coagulation cascade were seen in the present study, possible alterations in the complement and kininogen cascades have yet to be explored. Our results suggest that chronic inflammation of the laminae of the hoof might be associated with system-wide changes in immune function and that this hypothesis warrants further investigation. These conclusions are discussed in detail below.

### Technological considerations

DIGE has been used successfully to identify contributing factors and biomarkers of a number of human diseases, including multiple sclerosis [18], acute and chronic liver disease [19,20], and various cancers [21-25]. To our knowledge, however, DIGE has only been applied to horses in two previous studies; these authors used DIGE with and without depletion of abundant serum proteins to investigate equine recurrent uveitis (ERU) [26,27], a chronic inflammatory disorder of the eye with many similarities to human autoimmune uveitis. As in our study, antibodies directed against human proteins removed a large portion of abundant equine serum proteins, including albumin and IgG [27]. Zipplies et al. identified kininogen, apolipoprotein A-IV, IgM, and alpha-2-HS glycoprotein as differentially expressed in ERU [27]; the overlap between their results and ours may be because ERU and chronic laminitis are both chronic inflammatory diseases and similar pathways are likely involved in disease pathogenesis. It must be noted, however, that the direction of change seen in APOA-IV and kininogen in the present study was not consistent with that seen by Zipplies et al., although the reason for this remains unknown.

Of the 16 differentially expressed proteins identified in this study, 14 have already been associated with other chronic diseases in rats [28], humans [18-22,24,29,30], and horses [26] using DIGE. To our knowledge, coagulation factor X and IgA have not been associated with either acute or chronic diseases using DIGE based proteomics. The high level of redundancy in differentially expressed proteins among various disease states could result from the known bias of DIGE towards detecting relatively high abundance proteins. Alternatively, there could be a core group of proteins that are differentially expressed during chronic inflammation. Analogous to the “acute phase reactants” seen during acute trauma, infection, or stress, these “chronic phase reactants” might serve a similar purpose in modulation of the immune response. Further standardization of experimental proteomics methods and meta-analysis of a larger number of studies is necessary to confirm or refute this hypothesis.

### Coagulation and complement

In our study, the majority (11 of 16) of differentially expressed proteins belonged to the inter-related coagulation, complement, and kininogen cascades. The coagulation cascade in LMN horses was characterized by the decreased expression of clotting factors such as factor X and fibrinogen and the increased expression of the inhibitor antithrombin, all of which would inhibit clotting. This was balanced, however, by an increase in alpha-2-macroglobulin and a decrease in protein S; the effect of these two changes would be to promote clotting through the inhibition of the anti-coagulant protein C. Changes in abundance of liver-associated clotting proteins are dependent upon the rates of both production and consumption, so the DIGE results provided limited information about the ability of the blood to clot. We therefore assessed the functional significance of our results using a coagulation panel, which showed a slightly prolonged prothrombin (PT) time. It should be noted that, even though PT time was elevated above normal levels in 5 horses, the clinical relevance of this finding is yet to corroborated by other approaches. Findings from the coagulation panel were sufficient to rule out the presence of major coagulopathies such as disseminated intravascular coagulation and antithrombin deficiency, but unfortunately do not provide insight as to the mechanism underlying the changes in clotting proteins seen with DIGE. One possible explanation for the alteration in liver-associated proteins is the over-expression of interleukin-1 (IL-1) by white blood cells of horses with chronic laminitis (S. Steelman, unpublished observation). IL-1 has the potential to both positively and negatively regulate hepatocyte expression of a number of acute phase proteins [31]; specifically, it has been shown to induce expression of angiotensinogen and alpha-2-macroglobulin [32,33], both of which were upregulated in our study. Further investigation of this possibility is currently underway in our laboratory.

As two of the horses in the LMN group were being treated with the NSAID phenylbutazone at the time of sample collection, this had the potential to influence our results. Although, to the best of our knowledge, phenylbutazone use has not been proven to directly alter the expression of any of the diffferentially expressed proteins in the current study, we cannot rule out this possibility. In addition, NSAIDs are known to affect the coagulation cascade through their regulation of prostaglandins; however, we saw no obvious differences in any of the clotting parameters tested in the two horses receiving treatment (L1 and L2) as compared with the rest of the group. Indeed, previous reports suggest that phenylbutazone at the dose used in the current study had no effect on thrombus formation, fibrinogen concentration, or factor Xa activity in rats [34].

### APOA-IV

Our results showed a significant upregulation of apolipoprotein A-IV in horses with chronic laminitis as compared to controls. APOA-IV is an essential component of chylomicrons and is produced by the small intestine, particularly in response to the ingestion of triglycerides [35]. In this way, it functions as a satiety factor [36], but it is also known to have anti-oxidant [37] and anti-inflammatory [38] properties. Vowinkel and colleagues [38] showed that APOA-IV ameliorates experimentally-induced colitis in mice by attenuating the upregulation of P-selectin and decreasing microvascular adhesion of platelets and leukocytes. Studies in horses and dogs have shown extensive polymorphism of APOA-IV [17]; in humans, certain alleles have been associated with elevated blood glucose and triglyceride levels as well as body mass index [16]. The tissue sources and physiological functions of APOA-IV have yet to be explored in horses. Evidence exists, however, that serum APOA-IV levels are influenced by the gastrointestinal microflora in mice [39], which, like horses, are hindgut fermenters. Preliminary data from our laboratory suggest that the composition of fecal microflora is altered in horses with chronic laminitis as compared with controls, thus providing a possible explanation for the elevation of APOA-IV seen in the present study. The underlying mechanism is currently unknown, although the composition of the hindgut microflora play an important role in the development of acute laminitis [40] and could possibly contribute to exacerbation of symptoms in chronic laminitis as well. Future studies will focus on these questions and their relevance to the problem of equine laminitis.

## Conclusions

Our results suggest that chronic equine laminitis is associated with specific changes in the plasma proteome, which might be reflective of alterations in the immune system that are not confined to the foot. Specifically, the altered abundance of complement proteins and acute phase reactants suggests a chronic activation of the innate immune response. Our results provide a base upon which to build future studies with the ultimate goal of attenuating inflammation and reducing the pain associated with this devastating disease.

## Methods

### Animals and sample collection

Blood samples were collected from horses residing at the Hoof Diagnostic and Rehabilitation Clinic (Bryan, TX) in accordance with the Texas A&M University College of Veterinary Medicine guidelines regarding research performed on non university-owned animals. All animal protocols were approved by the Texas A&M University College of Veterinary Medicine Clinical Research Review Committee (CRRC# 10–52) and were performed with the consent of the owner or the owner’s agent. Horses were diagnosed as having chronic laminitis (LMN, n = 4) by a licensed veterinarian based on clinical presentation, case history, and radiographic evidence of dorsopalmar rotation of the distal phalanx. Horses in the control group (CON, n = 4) were housed either at the clinic or at a nearby farm and had no history of laminitis. Both groups of horses, CON and LMN, were 15 to 20 years of age. Please see Additional file 1 for detailed descriptions of each animal used in the study.

Blood samples were collected from the jugular vein into evacuated blood tubes containing either heparin or sodium citrate. Tubes were inverted a minimum of eight times to ensure adequate mixing of the anticoagulant. Heparinized samples were centrifuged at 1200 *x g* and plasma was harvested and stored at −80°C. No protease inhibitors were added to the samples. Citrated samples were immediately transported to the Texas A&M College of Veterinary Medicine Clinical Pathology Laboratory for evaluation of coagulation parameters. All samples were collected between 9:00 and 11:00 am.

### Sample preparation for DIGE

All sample preparation and DIGE experiments were conducted at the Texas A&M University Protein Chemistry Laboratory. Details of the DIGE methods used have been described previously [41]. Frozen plasma (CON, n = 4; LMN, n = 4) was depleted of albumin and IgG using human-specific affinity chromatography columns (Seppro, Sigma-Aldrich, St. Louis, MO) to enrich samples for low-abundance proteins. Human affinity chromatography reagents have been used successfully in horses for depletion of serum proteins [27]. A reference sample (containing equal amounts of protein from each of the 4 CON and 4 LMN samples) was used as a inter-gel standard. Forty-five micrograms of each CON and LMN sample was labeled with 200 pM Cy3 or Cy5 dye in a balanced design to reduce potential effects of dye bias; the reference sample was labeled with 200 pM Cy2. Equal amounts of paired LMN and CON samples, plus the reference sample, were mixed and loaded onto an isoelectric focusing strip (DryStrip, 24 cm, pH 4–7, GE Healthcare). Proteins were focused at 50 μA per strip for a total of 50,200 Vh and then separated on 12% SDS-PAGE gels. A total of four gels were used, with each gel containing equal amounts of protein from one CON sample, one LMN sample, and the reference sample.

### Gel imaging and DeCyder analysis

Gels were scanned on a Typhoon Trio fluorescent imager (GE Healthcare) with an image resolution of 100 μm. The Biological Variation Analysis (BVA) and the Batch Processor modules of the DeCyder v6.5 software package were used to identify protein spots in each gel, to quantify fluorescent intensity of the spots, and to perform statistics. Criteria for signficantly differentially expressed spots included identification on at least three of the four gels and a P value < 0.05 by Student’s independent *t* test. Spots selected for identification by LC-MS/MS were robotically picked (Ettan Spot Picker, GE Healthcare) out of one gel, digested with trypsin, and shipped to the University of Texas – San Antonio Proteomics Core service lab for LC-MS/MS analysis.

### Protein identification

MS/MS samples were analyzed using Mascot (Matrix Science, London, UK; version Mascot) and X! Tandem (The GPM, thegpm.org; version 2007.01.01.1). X! Tandem and Mascot were set up to search the NCBI database of nonredundant proteins (NCBInr_20091018.fasta, 9910686 entries) assuming the digestion enzyme trypsin. Scaffold (version Scaffold_3_00_04, Proteome Software Inc., Portland, OR) was used to validate MS/MS based peptide and protein identifications. Peptide identifications were accepted if they could be established at greater than 95.0% probability as specified by the Peptide Prophet algorithm [42]. Protein identifications were accepted if they could be established at greater than 99.0% probability and contained at least 2 identified peptides. Protein probabilities were assigned by the Protein Prophet algorithm [43]. Mass spectra have been deposited in the PRIDE database [44] under accession number 22842.

### Gene ontology

The horse genome, although sequenced, is partially annotated; human orthologs of the equine proteins were therefore used to search for significant gene ontologies. Biological processes, molecular functions, cellular components, and relevant pathways were identified using the DAVID algorithm (v6.7, [45-47]). Significance was judged by an adjusted P value less than 0.01 and a false discovery rate of less than 5%.

### ELISAs

Capture ELISAs were performed to validate DIGE results for selected proteins. Plasma concentrations of IgA and IgM were determined using validated antibody pairs (Bethyl Labs, Montgomery, TX) and standard protocols. Briefly, high protein-binding 96 well plates (Nunc MaxiSorp, Thermo Scientific, Rockford, IL) were coated with 100 μg of unlabelled capture antibody overnight at 4°C. Plates were blocked with SuperBlock buffer (Thermo Scientific, Waltham, MA) for 30 min at room temperature. Whole plasma samples were diluted 1:4,000 in assay buffer (10% SuperBlock in PBS) and 100 μl of each diluted sample was pipetted in duplicate onto the plates, which were then incubated at 37°C for 90 min. HRP-conjugated detection antibodies were diluted 1:50,000 (IgM) or 1:150,000 (IgA) in assay buffer and incubated on the plates for 2 h at room temperature. SigmaFast OPD (Sigma, St. Louis MO) was used as a substrate for HRP. Sample concentrations were calculated using dilutions of a reference serum of known concentration. Comparisons between groups were made by Student’s *t* test using a variation of the S programming language called R, which is commonly used for statistical analysis ( http://www.r-project.org). A P value < 0.05 was considered sufficient to reject the null hypothesis.

### Coagulation panel

In order to explore potential physiological consequences of differentially expressed proteins identified using DIGE, a standard panel of coagulation tests was performed by the Texas A&M College of Veterinary Medicine Clinical Pathology Laboratory on the 4 LMN horses used for the DIGE experiment plus an additional 3 LMN cases that were available at the time of sample collection. None of the CON horses were available for additional sample collection at this time. Reported equine-specific reference ranges are those used for clinical cases at the Texas A&M Veterinary Medical Teaching Hospital. Comparisons between groups were performed by χ^2^ analysis using the R programming language. A P value < 0.05 was considered sufficient to reject the null hypothesis.

### Western blot

Immunoblotting for APOA-IV was performed according to standard protocols. NuPage equipment, gels, and buffers (Invitrogen, Carlsbad, CA) were used for protein electrophoresis and transfer according to the manufacturer’s instructions. Briefly, 1 μl of plasma from each horse was mixed with 10x NuPage Sample Buffer, NuPage Reducing Agent, and 5.5 μl of water, heated to 70°C for 10 min, and separated by electrophoresis on 4-12% Bis-Tris gradient gels. Gels were transferred to PVDF membranes, which were then rinsed with distilled water and blocked using 5% non-fat dried milk (NFDM) in a Tris-buffered saline solution containing 0.05% Tween-20 (TBST). Primary antibody (mouse anti-human APOA-IV, Abcam, Cambridge, UK) was diluted 1:1,000 in NFDM-TBST. Secondary antibody (goat anti-mouse IgG – HRP conjugated, Abcam) was diluted 1:5,000 in NFDM-TBST. Bound antibody was visualized by addition of a chemiluminescent substrate (SuperSignal West Dura ECL, Thermo Scientific) and blots were imaged using a CCD camera system (Chemi-Doc, Bio-Rad, Hercules, CA). Densitometry was performed using Image J software [48]. Equal loading of all samples was confirmed by staining of the membrane for total protein using GelCode SafeBlue stain (Thermo Scientific). Comparisons between groups were made by Student’s *t* test. Please see Additional file 2 for a detailed description of the verification of antibody specificity and loading controls.

## Competing interests

The authors declare that they have no competing interests.

## Authors’ contributions

SMS designed the experiment, collected samples, performed the immunoassays and data analysis, and drafted the manuscript. BPC assisted in experimental design and data interpretation and critically reviewed the manuscript. All authors read and approved the final manuscript.

## Supplementary Material

Additional file 1 ** Table S1. **Details of individual animals used in the study. CON: control, LMN: laminitis, DIGE: animal was included in the DIGE proteomics experiment, COAG: animal was included in the coagulation panel experiment, WB: animal was included in the APOA-IV western blot experiment, G: gelding, M: mare, S: stallion, QH: Quarter horse or Quarter horse-type, TB: Thoroughbred or Thoroughbred-type, ARAB: Arabian, BCS: body condition score, ukn: unknown. Click here for file

Additional file 2 ** Table S2. **Equine proteins with similarity to human APOA-IV as determined by the NCBI BLAST algorithm. Only proteins with greater than 50% coverage are included. [49] Click here for file
