# Supplementary material for: Plasma proteomics shows an elevation of the anti-inflammatory protein APOA-IV in chronic equine laminitis
Source: BMC Vet Res. 2012 Sep 27;8:179. doi: 10.1186/1746-6148-8-179 (PMC3511297; doi:10.1186/1746-6148-8-179)
Supplement: Additional file 1 — Table S1. Details of individual animals used in the study. CON: control, LMN: laminitis, DIGE: animal was included in the DIGE proteomics experiment, COAG: animal was included in the coagulation panel experiment, WB: animal was included in the APOA-IV western blot experiment, G: gelding, M: mare, S: stallion, QH: Quarter horse or Quarter horse-type, TB: Thoroughbred or Thoroughbred-type, ARAB: Arabian, BCS: body condition score, ukn: unknown. [file 1746-6148-8-179-S1.pdf]

Steelman, S.M. and B.P. Chowdhary. Plasma proteomics shows an elevation of the anti-inflammatory protein APOA-IV in chronic equine laminitis.

Additional Information 1: Further description of study animals and sample collection

Details of individual horses are provided below in Supplemental Table 1. As many of these animals were resuced or donated to the Hoof Diagnostic and Rehbilitation Clinic, their exact age, breed, and cause of laminitis is unknown. Horses were maintained on a pelleted ration (12% protein, Producer’s Cooperative, Bryan, TX) and coastal bermudagrass hay. Samples were collected between the hours of 0700 and 1200 in the months of June and August, 2010.

None of the horses showed symptoms of pituitary pars intermedia dysfunction (PPID, although plasma ACTH levels were not specifically tested. In addition, none of the horses were obese, as assessed by body condition score. Total plasma protein was within the normal range and did not differ between groups, indicating that none of the study animals was dehydrated at the time of sample collection. Plasma levels of IL-1β were undetectable in both groups by equine-specific ELISA (data not shown).

Supplemental Table 1. Details of individual animals used in the study. CON: control, LMN: laminitis, DIGE: animal was included in the DIGE proteomics experiment, COAG: animal was included in the coagulation panel experiment, WB: animal was included in the APOA-IV western blot experiment, G: gelding, M: mare, S: stallion, QH: Quarter horse or Quarter horse-type, TB: Thoroughbred or Thoroughbred-type, ARAB: Arabian, BCS: body condition score, ukn: unknown.

Horse	Group	Experiment	Sex	Breed/Type	BCS
1	CON	DIGE, WB	G	QH	5
2	CON	DIGE, WB	M	QH	5
3	CON	DIGE, WB	G	TB	5
4	CON	DIGE, WB	S	QH	ukn
5	LMN	DIGE, COAG, WB	G	QH	5
6	LMN	DIGE, COAG, WB	G	TB	5
7	LMN	DIGE, COAG, WB	G	TB	5
8	LMN	DIGE, COAG, WB	M	ARAB	6
9	LMN	COAG, WB	M	ARAB	4
10	LMN	COAG, WB	G	QH	5
11	LMN	COAG	M	pony	6
