# Supplementary material for: Plasma proteomics shows an elevation of the anti-inflammatory protein APOA-IV in chronic equine laminitis
Source: BMC Vet Res. 2012 Sep 27;8:179. doi: 10.1186/1746-6148-8-179 (PMC3511297; doi:10.1186/1746-6148-8-179)
Supplement: Additional file 2 — Table S2. Equine proteins with similarity to human APOA-IV as determined by the NCBI BLAST algorithm. Only proteins with greater than 50% coverage are included. [49] [file 1746-6148-8-179-S2.pdf]

Steelman, S.M. and B.P. Chowdhary. Plasma proteomics shows an elevation of the anti-inflammatory protein APOA-IV in chronic equine laminitis.

Additional Information 2: Verification of APOA-IV western blot

The monoclonal anti-human APOA-IV antibody used in the present study detected an equine protein of ~28 kDa, which is smaller than the published molecular weight of human APOA-IV (46 kDa, [1]). We therefore performed a series of experiments to verify that the detected band was indeed APOA-IV. We first used NCBI's BLAST algorithm to determine the sequence similarity between human APOA-IV and the database of nonredundant proteins for *Equus caballus*. The match with the highest degree of similarity was equine APOA-IV (PREDICTED: apolipoprotein A-IV, XP_001502503.1), as shown in Supplemental Table 2 below. The next closest match was equine APOA-I (PREDICTED: apolipoprotein A-I-like, XP_001502519.1), although the percentage of matching amino acids was much lower (82% versus 26%). Although other proteins showed similar levels of homology with APOA-IV as did APOA-I, only APOA-I had a predicted molecular weight that corresponded with that of the observed band.

Supplemental Table 2. Equine proteins with similarity to human APOA-IV as determined by the NCBI BLAST algorithm. Only proteins with greater than 50% coverage are included.

Accession Number	Protein	E value	Max Identity (%)	Coverage (%)	Predicted MW (kDa)
XP_001502503.1	PREDICTED: apolipoprotein A-IV	0	82	98	45
XP_001502519.1	PREDICTED: apolipoprotein A-I-like	5.00E-25	26	91	28
XP_001500835.1	PREDICTED: apolipoprotein A-V-like	2.00E-24	29	86	41
XP_001503271.3	PREDICTED: laminin subunit alpha-2	5.00E-11	26	86	80
XP_001915354.1	PREDICTED: laminin subunit beta-1	0.016	23	68	198

To test the likelihood of the monoclonal antibody recognizing equine APOA-I and not APOA-IV, we determined the expression of both apolipoproteins at the RNA and protein levels in a panel of equine tissues (Supplemental Figure 1A and B). End point PCR showed that, whereas APOA-I was expressed in all tissues tested, APOA-IV was only present in the liver (Suppl. Fig 1A). A western blot of four tissues from the panel using the APOA-IV antibody detected a band in liver tissue, but not in brain or skeletal muscle (Suppl. Fig 1B), suggesting that the 28 kDa band was APOA-IV and not APOA-I. A very faint band was seen in heart tissue homogenate, likely due to blood present in the sample.

Based on these results, we included liver homogenate as a positive control on subsequent western blots (Suppl. Fig. 1C). A single band corresponding to the 28 kDa liver homogenate band was seen in all plasma samples. In addition, three different types of controls were performed to ensure equal loading in all lanes. First, the western blot was repeated in triplicate, as any technical variation in sample loading would be unlikely to be repeated on all three blots. Second, after probing for APOA-IV,

the membranes were washed and stained with GelCode Blue (Thermo Scientific, Wilmington, DE) to detect total protein. Quantitative results of APOA-IV levels in each sample were normalized to those of a ~150 kDa band on the GelCode-stained membrane. The 150 kDa band was chosen because it could be more reliably quantified than the albumin band, which tended to smear. Normalization of APOA-IV results to the 150 kDa band showed a 2.26-fold elevation in APOA-IV in horses with laminitis (CON 1.0 ± 0.33, LMN 2.26 ± 0.41, P = 0.03). Finally, APOA-IV results were normalized to the total protein concentration of each sample as determined by bicinchoninic assay (Pierce BCA Protein Assay Kit, Thermo Scientific). This method resulted in a 2.54-fold elevation in APOA-IV (P = 0.02), as shown in the main text (Fig. 2). Supplemental Figure 1C shows one of three technical replicate western blots with its corresponding 150 kDa band.

Supplemental Figure 1. Expression of APOA1 and APOA4 in various equine tissues was determined by PCR (A). (B) shows a western blot in which the APOA4 antibody detected a 28 kDa band only in liver tissue, consistent with PCR results for APOA4 but not APOA1. A very faint band was seen in myocardial tissue, likely due to contamination of the tissue with blood. (C) shows that the positive control APOA4 band detected in liver is the same molecular weight as that detected in serum. Blot in (C) is a technical replicate of the blot shown in Figure 2 of the main text. SM: skeletal muscle, H: heart, Li: liver, Br: brain, Lu: lung, K: kidney, Sp: spleen.

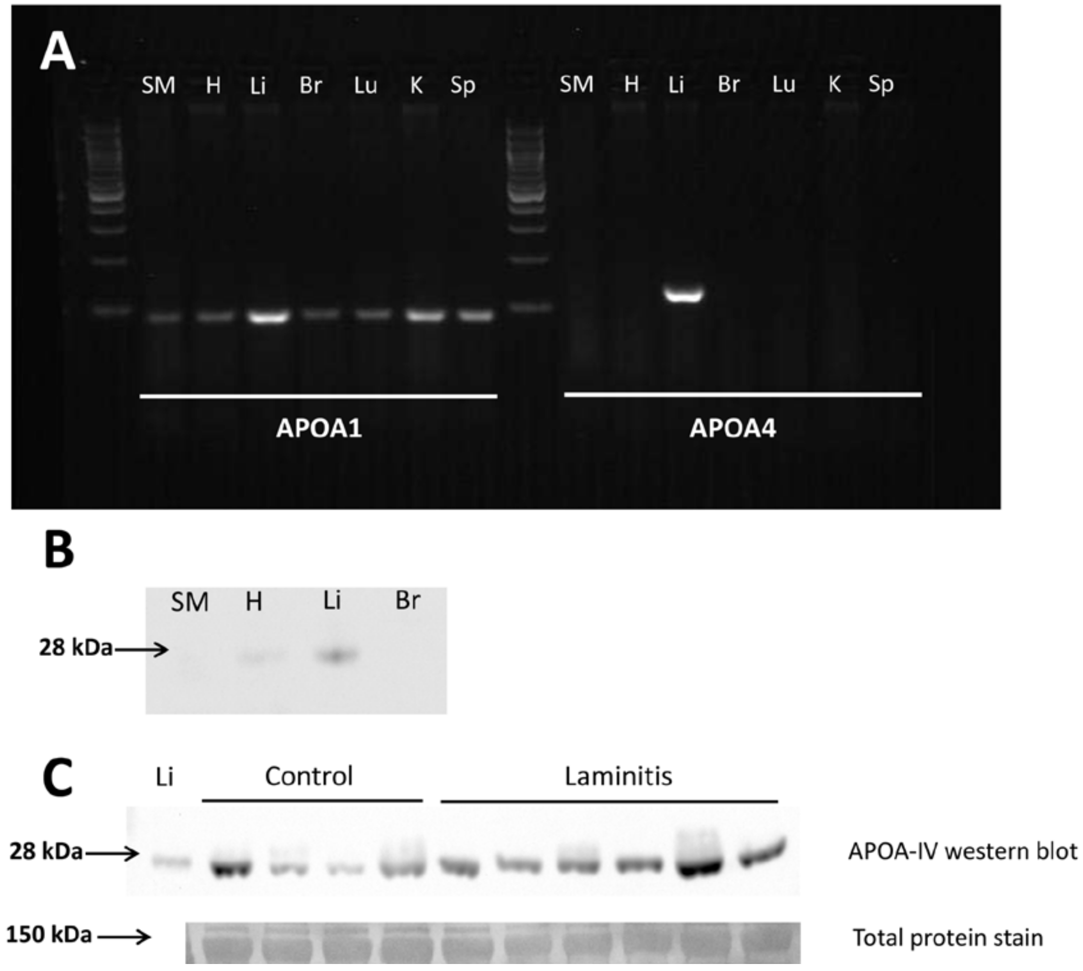

Reference

1. Beisiegel U, Utermann G: **An apolipoprotein homolog of rat apolipoprotein A-IV in human plasma. Isolation and partial characterisation**. *Eur J Biochem* 1979, **93**(3):601-608.
